# The Effect of Arsenic Trioxide and Its Combination with Oxaliplatin and Docetaxel on the Induction of Autophagy and Expression of LC3 and Beclin-1 Genes in AGS and MKN-45 Gastric Cancer Cell Lines

**DOI:** 10.34172/apb.42747

**Published:** 2024-12-05

**Authors:** Shadi Babaei, Mohsen Nikbakht, Ahmad Majd, Seyed Asadoullah Mousavi

**Affiliations:** ^1^Department of Biology, North Tehran Branch, Islamic Azad University, Tehran, Iran; ^2^Research Institute for Oncology, Hematology and Cell Therapy Tehran University of Medical Sciences, Tehran, Iran; ^3^Cell Therapy and Hematopoietic Stem Cell Transplantation Research Center, Tehran, Iran

**Keywords:** Gastric cancer, Autophagy, Apoptosis, Arsenic trioxide, Oxaliplatin, Docetaxel

## Abstract

**Purpose:**

Apoptosis and autophagy play critical roles in the survival and regulation of cancer cells, with key genes serving dual purposes in these processes. Arsenic trioxide (ATO), oxaliplatin (OXA), and docetaxel (DOC) are widely used in the treatment of various cancers. Specifically, ATO inhibits cellular proliferation and induces apoptosis in certain cancer cells, while OXA and DOC, common agents in cancer chemotherapy, continue to be actively studied for their potential therapeutic effects.

**Methods:**

This study investigated the effects of ATO, DOC, and OXA on AGS and MKN-45 gastric cancer cell lines in vitro. The MTT assay was utilized to determine the effective concentrations of these compounds, both individually and in combination. Apoptosis was assessed using Annexin V-FITC staining, and the mRNA levels of genes related to autophagy and apoptosis were analyzed via real-time polymerase chain reaction (PCR).

**Results:**

Our findings demonstrated that the combination of ATO with DOC and OXA significantly reduced the viability of AGS and MKN-45 cells compared to DOC or OXA therapy alone. Notably, the simultaneous administration of all three agents markedly enhanced apoptosis induction. Additionally, the combined use of two drugs showed a more pronounced impact on both cell necrosis and apoptosis compared to the effects of each drug used alone.

**Conclusion:**

The combination of two therapeutic agents represents a promising strategy for inducing autophagy and gene expression related to apoptosis in gastric cancer cells. This approach exerted a more substantial influence on cell apoptosis and necrosis than single-drug treatments, underscoring its potential as an effective therapeutic option.

## Introduction

 Gastric cancer ranks as the fifth most commonly diagnosed malignancy worldwide and constitutes the third leading cause of cancer-related mortality.^1^ Despite significant advances in therapeutic interventions, the overall 5-year survival rate for gastric cancer remains dismally low, hovering below 30%. This stark reality highlights an urgent imperative to develop more efficacious therapeutic modalities.^2^ The clinical heterogeneity of gastric cancer, reflected in diverse tumor aggressiveness, histopathological characteristics, and individual variations in therapeutic responses, further complicates treatment outcomes and necessitates innovative approaches to improve patient prognosis.

 A major obstacle to effective cancer therapy is the emergence of drug resistance, driven by complex cellular survival mechanisms, including autophagy.^3^ This highly conserved catabolic process is crucial for cellular homeostasis, involving the encapsulation of cytoplasmic components within double-membraned autophagosomes, which subsequently fuse with lysosomes to facilitate degradation and recycling.^4,5^ While autophagy serves as a tumor-suppressive mechanism by mitigating oxidative stress and maintaining genomic integrity in normal cells, paradoxically, it also functions as a survival mechanism in established malignancies, enabling cancer cells to withstand metabolic and therapeutic stressors.^6^ This dualistic nature makes autophagy a compelling target for therapeutic modulation, offering potential avenues to counteract resistance mechanisms in malignancies, including gastric cancer.^7^

 Arsenic trioxide (As₂O₃), with a medicinal history spanning over two millennia, gained approval from the U.S. Food and Drug Administration in 2000 for the treatment of acute promyelocytic leukemia.^8,9^ The antineoplastic properties of As₂O₃ are predominantly attributed to its capacity to induce apoptosis through the generation of oxidative stress, disruption of mitochondrial membrane potential, and activation of pro-apoptotic proteins.^10,11^ Beyond hematological malignancies, As₂O₃ has demonstrated notable efficacy in inhibiting angiogenesis, thwarting metastasis, and reversing chemoresistance in various solid tumors, including gastric carcinoma.^12,13^ These multifactorial effects render As₂O₃ a promising candidate for combinatorial strategies aimed at enhancing therapeutic efficacy and overcoming resistance barriers.

 In recent years, considerable interest has been directed toward the combinatorial potential of Arsenic trioxide (ATO) with cytotoxic agents such as docetaxel (DOC) and OXA to potentiate antitumor efficacy.^14,15^ DOC, a member of the taxane family, exerts cytotoxicity by stabilizing microtubules and disrupting mitotic function, demonstrating efficacy both as a monotherapy and in combination regimens.^16-20^ Conversely, OXA, a third-generation platinum analog, mediates its antitumor effects by forming DNA adducts and inhibiting DNA synthesis, distinguishing itself from cisplatin and carboplatin through a unique pharmacological profile and distinct toxicity spectrum.^21,22^

 This study seeks to elucidate the synergistic impact of combining ATO with DOC and OXA on autophagy-related gene expression and the induction of autophagy within AGS and MKN-45 gastric cancer cell lines, thereby offering novel insights into therapeutic strategies to mitigate resistance and improve clinical outcomes in gastric cancer.

## Materials and Methods

###  Chemicals and reagents

 A wide range of chemicals and reagents was utilized to ensure precise sample preparation and analysis. Fetal bovine serum (FBS), recombinant DMEM culture medium, penicillin/streptomycin, and Trypsin-EDTA were procured from Bio Idea Company (Tehran, Iran). Merck (Germany) supplied Trypan Blue, chloroform, isopropanol, and DMSO. Hemocytometer slides and MTT powder were sourced from TGI (Germany) and Sigma (Germany), respectively. Manual RNA extraction solutions and a cDNA synthesis kit were obtained from Genex Company (Iran), while DEPC-treated water was supplied by Cinacloon (Iran). Real-Time PCR Master Mix (2x) was provided by Amplicon Company (Denmark). All other reagents and materials, unless otherwise specified, were acquired from Merck or Sigma.

###  Cell culture 

 The MKN-45 and AGS human gastric cancer cell lines were obtained from the Pasteur Institute of Iran. The cells were consistently cultured in DMEM medium supplemented with 10% FBS. Adherent cells were sub-cultured at intervals of 2-3 days and subsequently harvested for further experimental procedures.

###  MTT assay for cell proliferation

 A solution was prepared with a concentration of 5 × 10⁴ cells/mL, and 200 µL of this cell suspension was added to each well of a 96-well culture plate. The plate was subsequently incubated at 37 °C in a 5% CO₂ atmosphere for 24 hours. Thereafter, 200 µL of MTT solution (0.5 mg/mL) was introduced to each well, and the cells were incubated for an additional 2-4 hours at 37 °C. Following this incubation, the solution was carefully removed, and 200 µL of DMSO was added to dissolve the formazan crystals formed within the cells. Optical density (OD) at 570 nm was then measured using a microplate reader.

###  RNA extraction and transcriptase-polymerase chain reaction (RT-PCR) assay

 RNA extraction was performed on homogenized samples using TRI pure reagent (Roche Applied Science, Germany) following the manufacturer’s protocol. An optimized procedure was applied with specific modifications to the initial sample processing. For the optimized protocol, a sample mass of 250 to 300 mg/mL of TRI pure reagent was used. The samples and reagent were transferred into microtubes and thoroughly homogenized until complete dissolution, then allowed to stand at ambient temperature for 10 minutes.

 The homogenized mixture was centrifuged at 4 °C at a relative centrifugal force of 12 200 × g for 10 minutes. The resulting supernatant was carefully transferred to a new microtube and combined with an equal volume of chilled isopropanol to facilitate RNA precipitation. After a 15-minute incubation, the mixture was centrifuged again at 12 200 × g for 15 minutes at 4 °C, yielding RNA-containing pellets. The supernatant was discarded, and the RNA pellets were washed with 1 mL of 75% ethanol at room temperature, followed by centrifugation at 4600 × g for 5 minutes. The supernatant was discarded, and the RNA pellets were air-dried for 10 minutes at 60 °C. The pellets were dissolved in DEPC-treated water, and RNA purity and concentration were measured using a NanoDrop ND-1000 spectrophotometer (Thermo, USA) at 260 nm. cDNA synthesis was performed from 1-2 µg of total RNA using a cDNA synthesis kit (Takara Bio Inc., Otsu, Japan) according to the manufacturer’s instructions. Real-time PCR amplification was conducted using the ABI Step One Plus^TM^ system (Applied Biosystems, USA) and gene quantification was performed on the Rotor-Gene Q system (QIAGEN, Hilden, Germany). Primer details are presented in Table 1.

**Table 1:** The primers for real-time PCR analysis

**Genes**	**Primers**	**Reference**
*Beclin-1*	Forward: 5’-AGCTGCCGTTATACTGTTCTG-3’ Reverse: 5’-ACTGCCTCCTGTGTCTTCAATCTT-3’	^23^
*LC3*	Forward: 5’-GATGTCCGACTTATTCGAGAGC -3’ Reverse: 5’-TTGAGCTGTAAGCGCCTTCTA-3’	^24^
*Caspase3*	Forward: 5'-GACTCTGGAATATCCCTGGACAACA-3' Reverse: 5'- AGGTTTGCTGCATCGACATCTG-3'	^25^
*BCL2*	Forward: 5’-CTGCACCTGACGCCCTTCACC-3’ Reverse: 5’-CACATGACCCCACCGAACTCAAAGA-3	^26^
*GAPDH*	Forward: 5’-TGAACGGGAAGCTCACTGG-3’ Reverse: 5’-TCCACCACCCTGTTGCTGTA-3’	^27^

###  Flow cytometry and cell cycle

 Flow cytometric analysis was employed to assess cell cycle distribution using the fluorescent dyes propidium iodide (PI) and Annexin-V. MKN-45 and AGS cells were cultured in 6-well plates for 36 hours, subsequently harvested, and fixed overnight at 4 °C in 70% ethanol to preserve cellular structure. Following fixation, cells were washed thoroughly with cold PBS and resuspended in a 400 µL staining solution containing PI, RNase A, and Triton X-100 to facilitate DNA staining and ensure optimal cell permeabilization. Incubation was carried out in the dark for 30 minutes at 37 °C. Additionally, Annexin-V staining was utilized for the detection and differentiation of apoptotic cells. Data acquisition was conducted using flow cytometry, and subsequent analysis of cell cycle phases (G0/G1, S, and G2/M) and apoptosis status was performed using FlowJo v7.6 software.

###  Statistical analysis

 The mean ± standard deviation was utilized to present the data, and GraphPad Prime software was utilized for data analysis. In order to evaluate the distinctions among various groups in different circumstances, the technique of analysis of variance (ANOVA) was utilized, along with Tukey’s multiple comparisons test. Statistical significance was determined by a *P* value < 0.05.

## Result

###  Cell viability evaluation using MTT test

**Figure 1:**
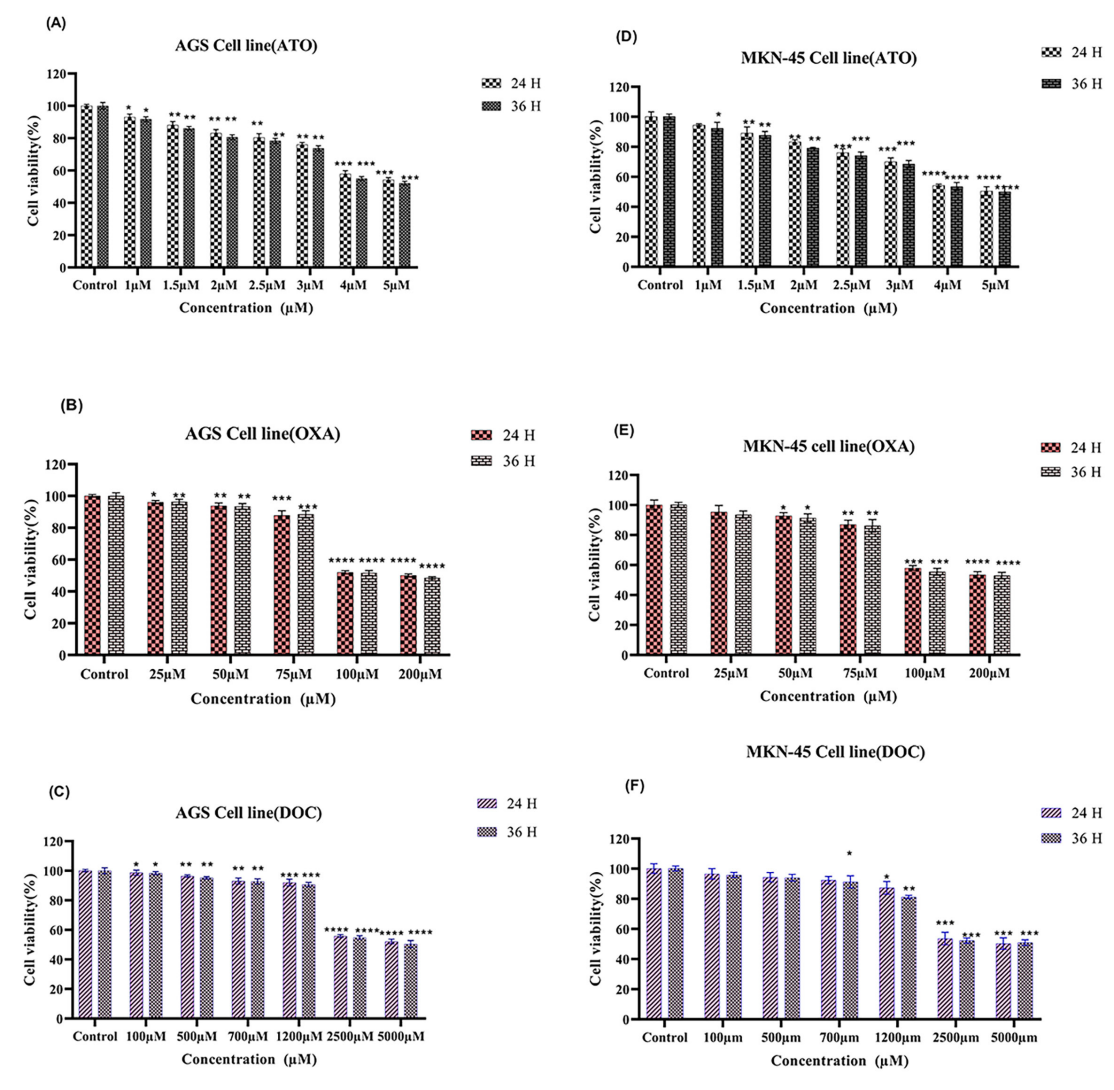
Cell viability was assessed in AGS and MKN-45 cell lines by MTT assay after treatment with (A) ATO, (B) OXA, and (C) DOC in AGS, and (D) ATO, (E) OXA, and (F) DOC in MKN-45 for 24- and 36-hours. The data, presented as mean ± SE of three independent experiments, were analyzed for statistical significance. The significance levels were denoted as * *P* < 0.05, ** *P* < 0.01, *** *P* < 0.001, and **** *P* < 0.0001 compared to the corresponding control. Additionally, the black star signified statistical significance in comparison to combination therapy, using two-way ANOVA

 The MTT assays demonstrated a dose- and time-dependent reduction in cell viability for ATO (4 µM), DOC (2500 µM), and OXA (100 µM) treatments. Figures 1A and 1D show that ATO significantly decreased viability in AGS and MKN-45 cells, with a more pronounced effect at 36 hours (*P* < 0.05). Figures 1B and 1E illustrate a similar dose-dependent decrease for DOC, with greater cytotoxicity at 36 hours. For OXA, Figures 1C and 1F indicate that higher concentrations led to reduced viability, particularly evident at 36 hours, with significant effects in both AGS and MKN-45 cells.

###  Cell viability ATO combination with OXA and DOC 

**Figure 2:**
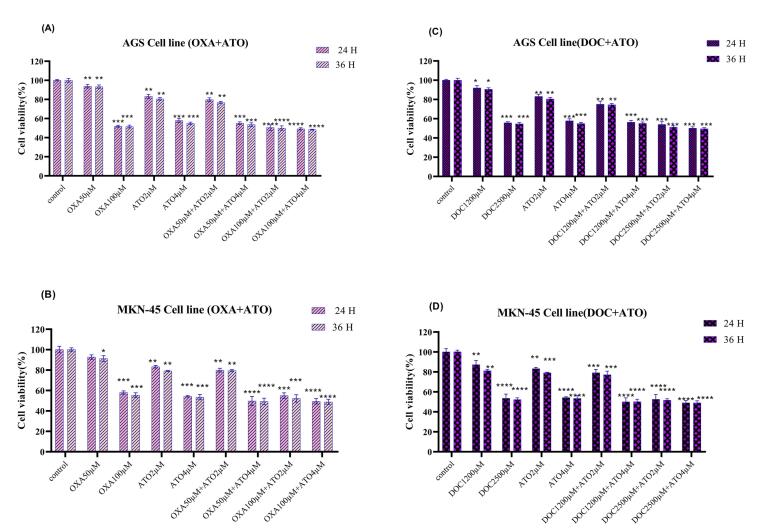
Cell viability. Effects of (A). ATO + OXA, (B). ATO + OXA in AGS and MKN-45, respectively. Effects of (C). ATO + DOC, (D)in AGS and MKN-45, respectively. The mean ± SE of three independent experiments were used to determine the statistical significance of the data obtained from the MTT assay after 24- and 36-hours treatment, with significance defined at * *P* < 0.05, ** *P* < 0.01, ***P < 0.001 and ****P < 0.0001 in comparison to the corresponding control.

 MTT assays demonstrated that combining ATO (4 µM) with OXA (100 µM) or DOC (2500 µM) significantly reduced cell viability in AGS and MKN-45 cells (Figures 2A-D). Both combinations exhibited greater cytotoxicity compared to single-drug treatments, with statistically significant reductions observed across different concentrations (*P* < 0.05). The effects were more pronounced after 36 hours of exposure compared to 24 hours.

###  Apoptosis assay

**Figure 3:**
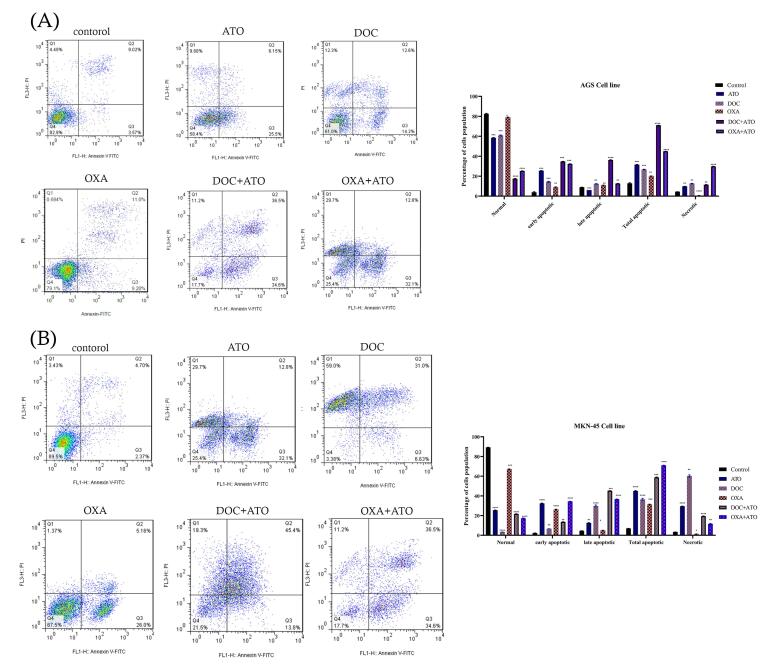
Analysis using flow cytometry. (A). Analysis of flow cytometry in AGS cells and (B). Analysis of flow cytometry in MKN-45 cells. AGS and MKN-45 cells treated with ATO, DOC, OXA, and their combination. Specifically, the lower left quadrant represents live cells, the upper left quadrant represents necrotic cells, the lower right quadrant represents early apoptotic cells, and the upper right quadrant represents late apoptotic cells. The obtained data were expressed as the mean ± SE of three independent experiments. The statistical significance was determined at *P < 0.05, **P < 0.01, ***P < 0.001, and ****P < 0.0001 in comparison to the corresponding control.

 The apoptotic cells were evaluated using flow cytometry after a 36-hour incubation period. The results of flow cytometry for AGS and MKN-45 cell line have shown that the drug leads to a reduce in the population of live cells (Q4) and an enhance in the population of early apoptotic cells (Q3), secondary apoptotic cells (Q2), and necrotic cells (Q1) following the drug’s effect on the AGS and MKN-45 cell line. As observed in Figures 3A and 3B, the percentage of AGS and MKN-45 cells undergoing apoptosis notably rose following drug administration (*P* < 0.05), in contrast to the control condition. The cells treated with ATO and OXA increased the proportion of apoptotic AGS and MKN-45 cell, with the effect of ATO + OXA being the most obvious. Compared with the control, drug treatment could significantly increase the incidence of apoptosis (*P* < 0.05) and a reduction in the population of viable cells occurs.

###  Cell cycle assay

**Figure 4:**
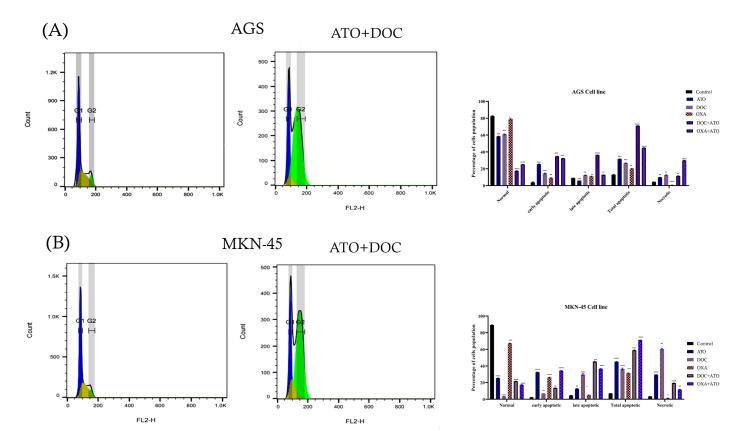
Cell cycle analysis. (A). Combination of ATO (4 µM) and DOC (2500 µM) in AGS (B. Effect of Combination of ATO and DOC that increased G2 for MKN-45.

 The effects of ATO combined with DOC on cell cycle progression demonstrated in Figure 4 (A and B). The findings show significant alterations in the distribution of cells across different phases of the cell cycle compared to untreated controls. In the AGS cell line, the control group exhibited an average of 4.82% of cells in the G0 phase. This proportion significantly decreased to 2.49% following treatment with ATO + DOC, suggesting a reduction in quiescent cells and potential promotion of cell cycle progression. In contrast, the G1 phase showed a decrease from 50.43% in the control group to 24.39% in the treated group, indicating that the combination therapy may disrupt the transition of cells into the G1 phase, resulting in a reduced cell population within this stage. A notable decrease was also observed in the S phase, with cell percentages dropping from 35.04% in the control group to 4.56% following treatment. This substantial reduction implies a strong inhibitory effect on DNA synthesis or cell cycle progression through the S phase, highlighting the treatment’s impact on cellular replication. Conversely, the G2 phase showed a marked increase, with cell percentages rising from 9.69% in the control group to 68.58% post-treatment, suggesting that the ATO + DOC combination may impede entry into mitosis, causing cell accumulation in this phase.

 Similarly, in the MKN cell line, the control group displayed an average of 8.30% of cells in the G0 phase, which decreased to 3.05% following ATO + DOC treatment, further indicating enhanced cell cycle progression due to the combined therapy. The G1 phase mirrored this trend, with a decline from 56.91% in the control group to 23.52% in the treated group, suggesting disrupted G1 phase transitions and a consequent decrease in the number of cells within this stage. The S phase also exhibited a reduction, from 27.81% in the control group to 10.69% in treated cells, reflecting significant inhibition of DNA synthesis and cell progression. Lastly, the G2 phase showed a pronounced increase, from 6.92% in the control group to 62.73% following treatment, indicating that the combination therapy effectively impedes progression into mitosis, leading to cell accumulation at this stage.

###  Real-time PCR assay

**Figure 5:**
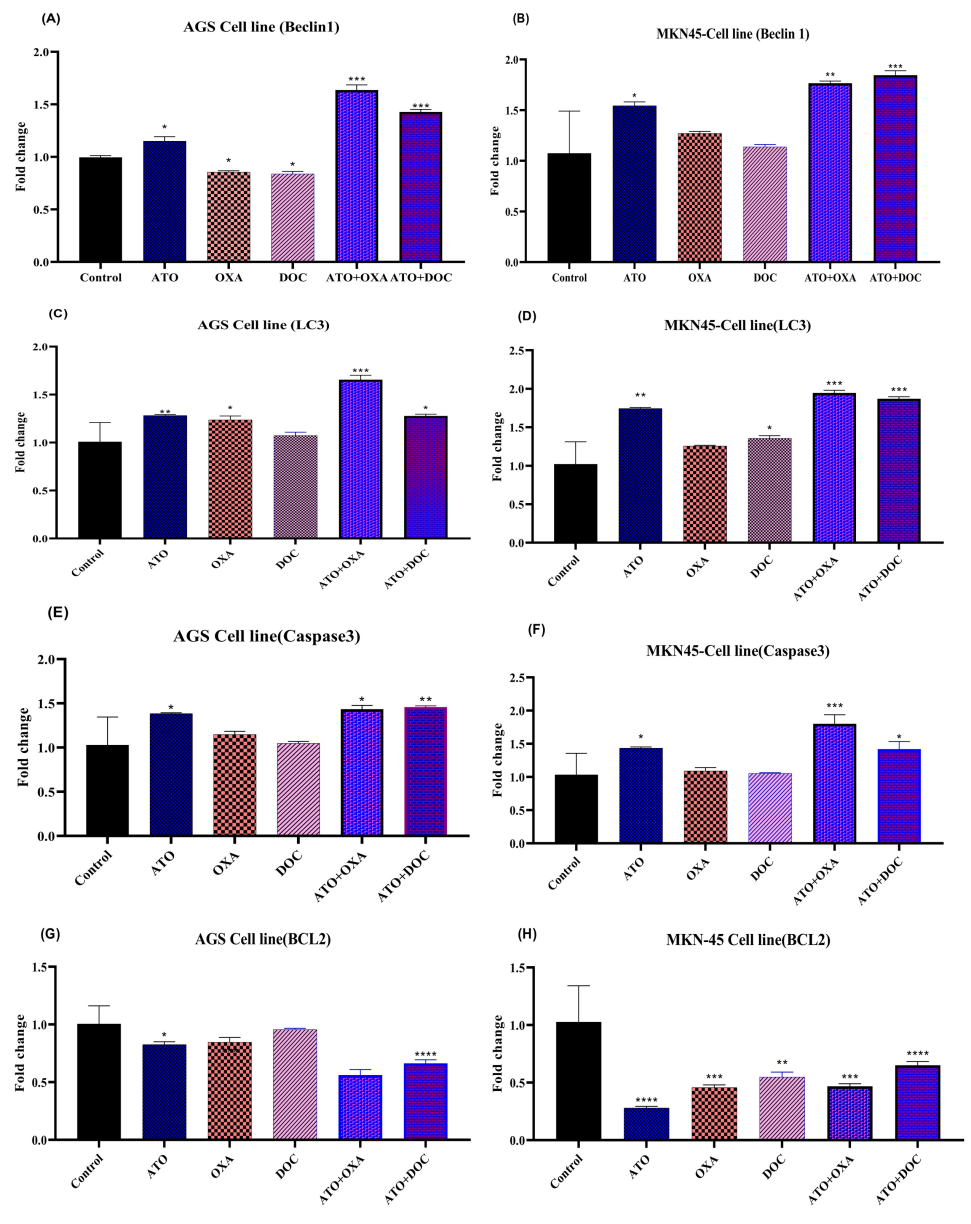
The effects of ATO, DOC, OXA and their combination on the mRNA level of indicated genes in AGS and MKN-45 cells. The effects of ATO, DOC, OXA on expression levels of cell proliferation genes (A, B) *Beclin-1*, (C, D), *LC3* (E, F) caspase3, and (G, H) BCL2 in AGS and MKN-45, respectively. The determination of values was accomplished through the utilization of Realtime PCR analysis. The mean ± SE of three independent experiments was provided for each value. In order to determine the statistical significance, a threshold of * *P* < 0.05, ** *P* < 0.01, *** *P* < 0.001, and **** *P* < 0.0001 was established, with comparisons made to the corresponding control.

 To investigate the molecular basis for the observed synergy of ATO, DOC, OXA, and their combinations, we performed real-time PCR to assess the expression of *Beclin-1*, *LC3*, *Caspase 3*, and *BCL2* in AGS and MKN-45 cells. As shown in Figures 5A and 5B, *Beclin-1* expression was significantly upregulated in the ATO-treated group compared to the control, while OXA and DOC treatments alone led to decreased expression. Combinations of ATO with either OXA or DOC induced higher *Beclin-1* expression compared to individual treatments. Figures 5C and 5D depict *LC3* gene expression across different treatment groups. In AGS cells, significant upregulation was observed with ATO and OXA treatment, while in MKN-45 cells, ATO and DOC combinations showed greater increases. Combined treatments of ATO with OXA or DOC produced the highest *LC3* expression levels relative to controls.

 The expression of *Caspase 3*, presented in Figures 5E and 5F, was significantly elevated in AGS cells treated with ATO, showing a 1.38-fold increase compared to controls. In contrast, OXA and DOC alone resulted in modest increases (1.14-fold and 1.04-fold). The combination of ATO with either OXA or DOC led to greater increases (1.43-fold and 1.45-fold, respectively). In MKN-45 cells, ATO treatment alone induced a 1.43-fold rise in *Caspase 3* expression, though not statistically significant (*P* < 0.05). The combination of ATO with OXA produced a 1.92-fold increase, while ATO with DOC led to a 1.51-fold increase, surpassing individual drug effects.

Figures 5G and 5H illustrate *BCL2* expression, which decreased significantly in ATO-treated AGS cells (0.82-fold reduction), while OXA and DOC showed modest decreases. Combinations of ATO with OXA or DOC further reduced *BCL2* levels. In MKN-45 cells, significant decreases were observed across all treatments, with combined therapies showing the greatest reductions, emphasizing a shift towards pro-apoptotic signaling.

## Discussion

 The development of cancer is characterized by aberrant gene expression, driven by genetic mutations and epigenetic alterations, ultimately leading to unregulated cell proliferation and resistance to therapy,^28^ This study explored the in vitro efficacy of ATO, DOC, and OXA, individually and in combination, in AGS and MKN-45 cell lines. By analyzing the modulation of key regulatory molecules such as *Beclin-1*, *Caspase 3*, *LC3*, and *BCL2*, we aimed to elucidate their effects on apoptotic and autophagic pathways. While apoptosis is a tightly regulated process of programmed cell death that eliminates damaged or unwanted cells, autophagy serves as a cytoprotective mechanism, facilitating cellular adaptation through the sequestration and degradation of intracellular components under stress condition.^29,30^

 Extant literature has established ATO as a potent inducer of autophagy, notably within leukemia cells under both in vitro and in vivo conditions.^31^ Nevertheless, our findings contrast with this paradigm, demonstrating that ATO, when combined with DOC and OXA, predominantly induces apoptosis rather than autophagy within gastric cancer cells. This divergence underscores the unique mechanistic interplay that favors apoptotic pathways when these agents are administered concurrently. Previous studies have illuminated ATO’s capacity to drive apoptosis and cellular differentiation in hematopoietic and solid tumors, yet our observations reveal a novel therapeutic avenue through its combinatorial efficacy with DOC and OXA,^32^ In support of this, previous work has shown that ATO, when used alongside sorafenib, significantly increases apoptotic responses and modulates key autophagy markers in leukemia cell lines, further highlighting its versatility as an anti-cancer agent.^26^

 In corroboration, Jia et al. elucidated that autophagy inhibition augments the chemosensitivity of castration-resistant prostate cancer (CRPC) cells, potentiating tumor cell death through apoptotic mechanisms.^33^ Their findings suggest that autophagy inhibition may represent a strategic approach to mitigating chemotherapy resistance in malignancies. Specifically, their investigation demonstrated that DOC treatment, coupled with IL-6-mediated modulation of the *LC3-II/LC3-I* ratio and an elevation in p-STAT3 levels, reflects the negative regulation of autophagy by p-STAT3. Our study resonates with these observations, indicating that the inhibition of autophagy sensitizes cancer cells to chemotherapeutic regimens, thereby intensifying apoptotic responses.^34^ Indeed, our data reveal that DOC potentiates the pro-apoptotic effects of ATO by upregulating *Caspase 3* expression and downregulating *BCL2*, thereby shifting the cellular balance towards apoptosis-driven cell death.

 Consistent with these observations, we found that DOC enhances ATO-induced apoptosis by modulating key apoptotic pathways. Research indicates that OXA can induce both autophagy and apoptosis depending on the cellular context.^34^ While previous studies primarily focused on OXA as a monotherapy, our findings highlight the synergistic apoptotic potential of its combination with ATO. The interplay between OXA-induced DNA damage and ATO’s modulatory influence on apoptosis-related pathways, such as p53 regulation and PML-RARα signaling, exemplifies a complex, multifaceted strategy for promoting cell death in resistant gastric cancer cells.^35^

 The inhibition of DOC-induced autophagy through pathways such as STAT3 activation has been documented to precipitate heightened mitochondrial dysfunction and a decline in cell viability.^36^ Our study similarly revealed that DOC accentuates ATO-driven apoptosis by modulating pivotal apoptotic pathways. Additional evidence indicates that inhibitors targeting pathways such as PI3K-AKT-mTOR display limited efficacy in isolation but achieve significantly enhanced cytotoxicity when co-administered with agents like ATO.^37^ Moreover, previous investigations have demonstrated that autophagy inhibitors, such as HCQ and BafA1, potentiate the cytotoxic and apoptotic effects of ATO and ATRA in leukemia cells, underscoring the potential of autophagy modulation in enhancing therapeutic outcomes.^38^ In alignment with these findings, our data demonstrate that ATO disrupts BCL2 protein homeostasis, induces mitochondrial dysfunction, and activates Caspase 3 when combined with DOC and OXA, thereby reflecting a sophisticated interplay between autophagic and apoptotic regulatory mechanisms.

 Moreover, the interplay between OXA-induced DNA damage and ATO’s modulation of p53 and PML-RARα pathways further underscores the intricate regulation of apoptosis. Our data illustrate that the synergistic interaction between OXA and ATO modulates BCL2 expression and activates Caspase 3, thereby promoting the intrinsic apoptotic cascade. While OXA exerts its influence through p53-mediated transcriptional regulation, ATO modulates nuclear structural dynamics, thereby amplifying apoptotic signaling.^39^ This comprehensive interaction accentuates the potential of combinatorial therapeutic strategies to overcome resistance mechanisms in gastric cancer.

## Conclusion

 This study establishes that ATO is a potent inducer of apoptosis and an effective inhibitor of cancer cell proliferation, with significant enhancements observed when combined with OXA and DOC. The synergistic interactions led to a marked reduction in gastric cancer cell populations, highlighting ATO’s capacity to amplify the cytotoxic effects of DOC and OXA. Cell cycle analyses confirmed that the combination therapy inhibited cancer cell growth and division while maintaining a balance between apoptotic and autophagic pathways. Gene expression analyses further demonstrated that these agents, in the presence of ATO, effectively drive programmed cell death. Future research should investigate the oxidative status of treated cells to further elucidate the underlying mechanisms of autophagy and apoptosis, thereby providing deeper insights into the therapeutic potential of combination strategies in cancer treatment.

## Competing Interests

 The authors declare no conflict of interest.

## Ethical Approval

 Not applicable.
